# *Solidago canadensis* L. Essential Oil Vapor Effectively Inhibits *Botrytis cinerea* Growth and Preserves Postharvest Quality of Strawberry as a Food Model System

**DOI:** 10.3389/fmicb.2016.01179

**Published:** 2016-08-02

**Authors:** Shumin Liu, Xingfeng Shao, Yanzhen Wei, Yonghua Li, Feng Xu, Hongfei Wang

**Affiliations:** Department of Food Science and Engineering, Ningbo UniversityNingbo, China

**Keywords:** *Solidago canadensis* L., *Botrytis cinerea*, strawberry, quality, gray mold, induced disease resistance

## Abstract

This study investigated the anti-fungal properties of *Solidago canadensis* L. essential oil (SCLEO) against *Botrytis cinerea in vitro*, and its ability to control gray mold and maintain quality in strawberry fruits. SCLEO exhibited dose-dependent antifungal activity against *B. cinerea* and profoundly altered mycelial morphology, cellular ultrastructure, and membrane permeability as evaluated by scanning electron microscopy, transmission electron microscopy, and fluorescence microscopy. SCLEO vapor at 0.1 mL/L maintained higher sensory acceptance and reduced decay of fresh strawberry fruit, and also reduced gray mold in artificially inoculated fruit. SCLEO treatment did not, however, stimulate phenylalanin ammonia-lyase, polyphenol oxidase, or chitinase, enzymes related to disease resistance. This suggests that SCLEO reduces gray mold by direct inhibition of pathogen growth. SCLEO vapor may provide a new and effective strategy for controlling postharvest disease and maintaining quality in strawberries.

## Introduction

Strawberries are in high demand because of their delicious flavor and nutritional value, but often become unmarketable due to mechanical injury and fungal contamination. Gray mold, caused by the fungus *Botrytis cinerea*, is one of the major causes for the reduced post-harvest storage life of strawberries (Lazar et al., 2010). In past decades, chemical fungicides were widely used to control postharvest fungal disease in fruit. However, the indiscriminate and excessive use of synthetic fungicides has been a prime cause for the development of resistant fungal pathogen populations, resulting in the use of even greater quantities of antifungal compounds in agriculture and the appearance of increased levels of toxic residues in food products (da Cruz Cabral et al., 2013). Alternative control methods are therefore urgently needed (Sukorini et al., 2013). Plant essential oils (EOs) and extracts have been used for thousands of years in food preservation, pharmaceuticals, alternative medicine and natural therapies (Prabuseenivasan et al., 2006). EOs, which are naturally synthesized in different plant organs as secondary metabolites, are characterized as oily fragrant liquids extracted from aromatic plant materials (El Asbahani et al., 2015). EOs have recently attracted interest as control agents for postharvest disease due to their volatility, relative safety, broad acceptance by consumers, and eco-friendly and biodegradable properties (Tzortzakis and Economakis, 2007). Numerous studies have documented antifungal effects for different EOs used to control deterioration in postharvest fruit such as citrus (Fan et al., 2014; Tao et al., 2014a; Shao et al., 2015), strawberry (Shao et al., 2013a), blueberry (Mehra et al., 2013), peach (Elshafie et al., 2015), and cherry tomato (Guerra et al., 2015).

*Solidago canadensis* L. is an herbaceous perennial of the family Asteraceae that is widely distributed in South America, Europe, and Asia (Skrzypczak and Budzianowski, 2001). It was intentionally introduced to eastern China as an ornamental plant in 1913 (Jin et al., 2004). Since the 1980s, *S. canadensis* has spread rapidly and has become one of the most destructive invasive weeds in southeastern China (Guo and Fang, 2002). It has significantly reduced the abundance and diversity of native plant communities, and poses a growing threat to important ecosystems and regional economies (Guo, 2005). Questions concerning potential control strategies and/or possible uses for the plant are now of great interest. *S. canadensis* has been used in European phytotherapy for 700 years for the treatment of chronic nephritis, cystitis, urolithiasis, rheumatism, and as an antiphlogistic drug (Apati et al., 2003). Its leaves contain a wide range of active ingredients that are responsible for its antioxidant, antimicrobial, anti-inflammatory and spasmolytic and diuretic properties (Wang et al., 2006; Deng et al., 2015). α-Pinene, germacrene D, and 6-*epi*-β-cubebene are the major components of the EO found in leaves from several *Solidago* species (Kalemba et al., 1990; Kasali et al., 2002; El-Sherei et al., 2014). These compounds may contribute to the antibacterial ability observed against *Listeria monocytogenes*, *Staphylococcus aureus* (Deng et al., 2015), and *B. cinerea* (Wang et al., 2006). Importantly, acute toxicity assays show that the *S. ÿanadensis* extracts have no obvious toxicity (Nie et al., 2008).

To the best of our knowledge, *Solidago canadensis* L. essential oil (SCLEO) has not yet been applied to strawberry fruit during storage. The aims of this study were to (1) investigate the effects and possible mechanisms of SCLEO treatment against *B. cinerea in vitro*, (2) examine effects of SCLEO treatment on the postharvest quality of fresh strawberry, and (3) measure the induction of disease resistance, and assess the control of gray mold, in artificially inoculated fruit treated with SCLEO vapor.

## Materials and Methods

### Essential Oil, Pathogen, and Fruit

Leaves of fresh *Solidago canadensis* L. were collected in October 2014, dried in the dark at room temperature, and then powdered with a pulverizer. EO was isolated by hydro-distillation using a distillation apparatus and a mixture of approximately 150 g fresh leaves in 1800 ml distilled water. Subsequently, EOs were dried using anhydrous sodium sulfate, filtered, and stored in amber flasks (4°C) until tested. The oil obtained from the plant species possessed a iridescent coloration and a characteristic odor.

Highly virulent *B. cinerea* was isolated from spoiled, greenhouse-raised strawberry fruit. Isolate identity was confirmed using morphological and molecular criteria (Shao et al., 2013a). Fungal cultures were maintained on potato dextrose agar (PDA) medium at 25°C. Spore suspensions were harvested from 10 day old cultures and adjusted to 1 × 10^6^ spores/mL by hemocytometer.

Strawberries (*Fragaria ananassa* Duch. cv. Hongyan) were harvested by hand at the mature red stage from a commercial greenhouse near Ningbo University, PR China, and transferred to the laboratory within 1 h. All fruit used in experiments were uniform in size and free of defects.

### Effects of SCLEO on Mycelial Growth

The toxicity of the SCLEO against *B. cinerea* was assessed using the method of Shao et al. (2013a). Plates were subjected to different SCLEO vapor concentrations (0.5, 1, 2, and 3 mL/L air) and then incubated at 25°C for 3 days. Treatment efficacy was evaluated by measuring and averaging two perpendicular diameters for each colony. Mycelial inhibition rate = (dc-dt)/(dc-di) × 100, where dc is the mean colony diameter of the control sets, dt is the mean colony diameter of the treatment sets, and di is the initial colony diameter of fungal PDA disks. All tests were repeated five times.

### Effects of SCLEO on Fungal Morphology and Ultrastructure

One hundred and fifty milliliter potato dextrose broth (PDB) medium was inoculated with 1 mL *B. cinerea* spore suspension (10^6^ spores/mL) and incubated at 25°C with shaking at 150 revolutions per minute (rpm) for 72 h. SCLEO was then added to the medium to a final concentration of 16.5 mL/L, and incubation continued for 2 h before samples were collected. Cultures without oil were used as controls. Samples were centrifuged at 4000 rpm for 10 min and washed with cold phosphate buffer solution (PBS, 0.1 M, pH = 7.4) three times collect fungal mycelia. Mycelia were fixed with 2.5% glutaraldehyde for 2 h at 4°C. Three replicates were prepared for the treated and control groups. The effects of SCLEO on hyphal morphology and cell ultrastructure of *B. cinerea* were observed by scanning electron microscopy (SEM) and transmission electron microscopy (TEM), using our previously described methods (Yu et al., 2015).

### Effects of SCLEO on Fungal Membrane Integrity

Membrane integrity was assayed by fluorescent microscopy (FSM) method, following the method of Liu et al. (2010). *B. cinerea* was treated with 16.5 mL/L SCLEO and collected as described in Section “Effects of SCLEO on Fungal Morphology and Ultrastructure”. The collected mycelium was stained with 50 mg/L propidium iodide (PI) for 30 min at 4°C in the dark. Residual dyes were removed by washing twice with phosphate buffered saline. Samples were observed with a Zeiss Axioskop 40 microscope (Carl Zeiss, Oberkoch en, Germany) equipped with a single fluorescein rhodamine filter set (Zeiss no.15: excitation BP 546/12 nm, emission LP 590 nm). fields of view from each cover slip were chosen randomly, and all experiments were repeated three times.

### Effects of SCLEO on the Postharvest Quality of Fresh Strawberry Fruit

Fresh strawberries were divided into two groups. For the treated group (SCLEO), fruits were placed into 8 L polystyrene containers with snap-on lids and minitype shelves. A 20 W heater was placed in the container and 800 μL SCLEO was pipetted into a glass dish (80 mm diameter), placed on the heater, and then the fruit container was immediately sealed. The heater was powered for 30 min to promote oil volatilization before being turned off. The SCLEO concentration in the chamber was regarded as 0.1 mL/L air, which is the ratio of SCLEO volume (800 μL) to the container volume (8 L). The container remained sealed for 12 h and was maintained at 25°C. Samples without EO treatment were used as controls. After treatment, all strawberry fruits were removed from the sealed containers, and then stored at 20°C for 4 days. Each day, 15 fruits were randomly selected for sensory evaluation and to assess changes in weight, firmness, total soluble solids (TSS), and titratable acidity (TA) content. Each group was replicated three times and the entire experiment was performed twice.

#### Quality Measurements

Weight loss was expressed as the reduction in weight as a percentage of total weight. Fruit firmness was measured by a hand penetrometer (GY-1, Hangzhou Top Instrument Co., LTD, China) and expressed in Newtons (N). Soluble solid content (TSS) and titrated acid (TA) were measured by the method of Shao et al. (2012).

At the end of storage, the extent of decay was evaluated by decay index. Decay was evaluated visually using 10 fruit per replicate according to a 4-level scale, where 0 = no decay; 1 = slight decay, covering <25% of the fruit surface; 2 = moderate decay, covering >25% but <50% of the fruit surface; 3 = severe decay, covering >50% of the fruit surface. The decay index was calculated using the following formula: [(1 × N1 + 2 × N2 + 3 × N3 + 4 × N4) × (4 × N)], where N is the total number of fruit measured and N1, N2, N3, and N4 are the number of fruit showing the different degrees of decay (Cao et al., 2010).

#### Sensory Evaluation

Sensory profiles were assessed using a twenty-category scale (1-5 = dislike extremely, 6-10 = neither like nor dislike, 11-15 = like, 16-20 = like extremely) for color, aroma and decay. The approach is modeled after the method described by Gol et al. (2013). Ten panelists were trained at the beginning of the experiment to evaluate relevant fruit characteristics. Sensory tests were conducted in a sensory laboratory equipped with individual sensory compartments.

### Effects of SCLEO on Gray Mold and the Induction of Disease Resistance in Artificially Inoculated Strawberries

Strawberries with no physical defects were surfaced-disinfected with 75% ethanol and air-dried for two hours. A single artificial wound (depth 2 mm) was made in each fruit using a nail 2 mm in diameter then 15 μL of *B. cinerea* suspension (10^6^ spores/mL) was inoculated into each wound. Inoculated fruits were randomly divided into control and SCLEO-treated groups. SCLEO treatment was as described in section of 2.5. Following incubation, the decay index was measured.

To evaluate the induction of active defense responses by SCLEO treatment, tissue samples (1 cm distance from the edge of the wound or decay area) of ten fruits from each replicate were collected at 0, 12, 24, 36, 48, 60, and 72 h after inoculation. Samples of each time point from each replicate were mixed and frozen immediately in liquid nitrogen, and then stored at –80°C. All enzyme extraction procedures were conducted at 4°C. Phenylalanin ammonia-lyase (PAL) was extracted with 0.1 M PBS (pH 8.8) containing 5 mM β-mercaptoethanol and 2% polyvinyl polypyrrolidone (PVPP, m/v). polyphenol oxidase (POD) was extracted with 200 mM PBS (pH 6.4) and 2% PVPP. Chitinase (CHI) and β-1,3-Glucanase were extracted with 0.05 M sodium acetate buffer solution (pH 5.0) with 2% PVPP (m/v). All extracts were homogenized and centrifuged at 10,000 *g* at 4°C for 20 min. The supernatants was used for the assay.

Phenylalanin ammonia-lyase activity was analyzed using the method of Sellamuthu et al. (2013). One unit of PAL activity was defined as the increase of absorbance by 0.01 units per hour. POD activity was assayed as described by Kochba et al. (1977). One unit of POD activity was defined as the increase of absorbance by 0.001 units per min at 470 nm. β-1,3-Glucanase activity was determined using the method of Abeles et al. (1971). One glucanase unit catalyzes the creation of 1 mg of glucose per hour. CHI activity was measured using the method of Boller et al. (1983). One CHI unit catalyzes the creation of 1 μmol *N*-acetyl-D-glucosamine per hour. The specific activity of all enzymes was expressed as units per gram (U/g).

### Statistical Analyses

SAS Software (Version 8.2; SAS Institute, Cary, NC, USA) was used to conduct statistical analyses. Data were analyzed by one-way analysis of variance (ANOVA). Comparison of means was performed by Tukey’s HSD. The threshold for statistical significance was *P* < 0.05.

## Results

### Effects of SCLEO Vapor on Mycial Growth

**FIGURE 1:**
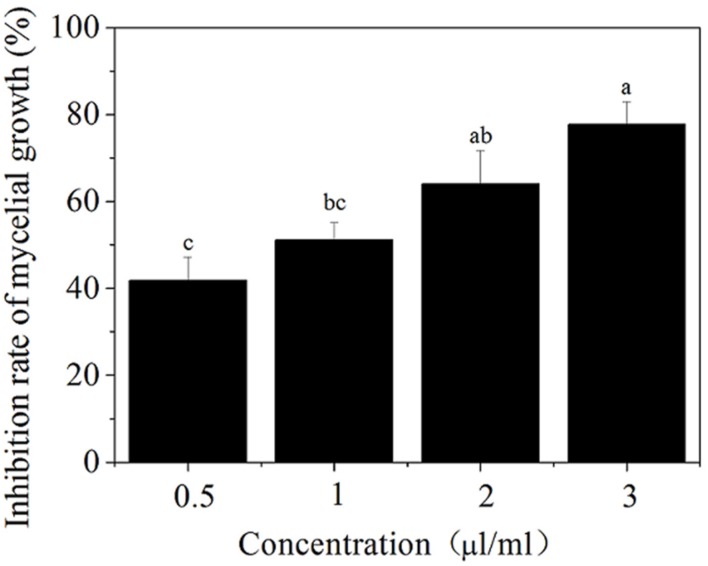
**Effects of *Solidago canadensis* L. essential oil (SCLEO) vapor at different concentration on mycelial growth of *Botrytis cinerea.*** Vertical bars represent the standard error of the means. Bars labeled with the same letter are not significantly different.

The antifungal ability of SCLEO to *B. cinerea* was observed in **Figure 1**. SCLEO inhibited the mycelial growth of *B. cinerea* in a dose-dependent manner (**Figure 1**). The lowest concentration (0.5 mL/L air) showed moderate antifungal activity against *B. cinerea* but inhibited nearly half of the mycelial growth (42%). Inhibition rate reached 78% at 3 mL/L of SCLEO.

### Effect of SCLEO Treatment on Hyphal Morphology and Ultrastructure

**FIGURE 2:**
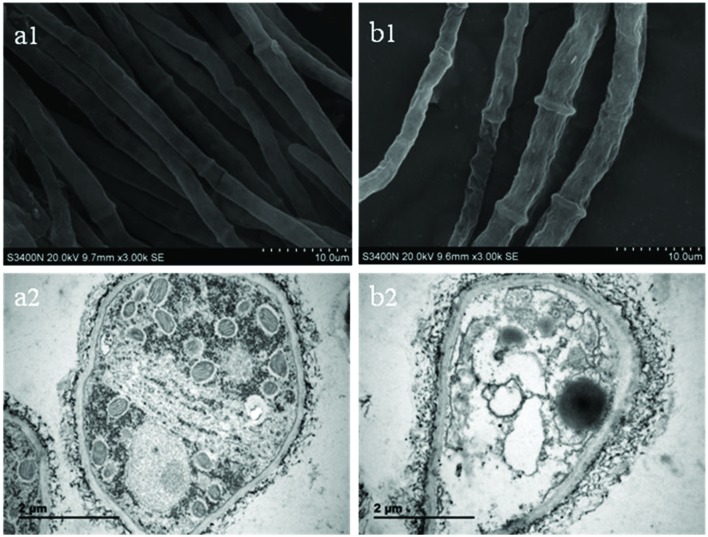
**Images obtained by scanning electron microscopy (SEM) and transmission electron microscopy (TEM) for *B. cinerea* treated with SCLEO**. First row: SEM images (×3000) of morphology in hyphae. **(a1)** Healthy hyphae control. **(b1)** hyphae treated with SCLEO. Second row: TEM images (×25000) of hyphal ultrastructure. **(a2)** Healthy hyphae control. **(b2)** Hyphae treated with SCLEO.

**Figure 2** shows morphological changes observed in *B. cinerea* treated with SCLEO. SEM examination of untreated hyphae showed regular and homogeneous morphological features; smooth dense surfaces with clearly defined septa and dense reticulation (**Figure 2a1**). In contrast, the morphology of treated *B. cinerea* hyphae is irregular, with slender and shriveled surfaces, enlarged septa and sparse reticulation (**Figure 2b1**). TEM examination in control samples revealed a typical hyphal structure; uniformly thick cell walls and clearly visible nuclei, endoplasmic reticulum, and mitochondria, as well as a dense matrix adhering to the intact membrane and cell wall (**Figure 2a2**). After treatment with SCLEO, severe structural damage was observed, including disrupted cell membranes, plasmolysis, leakage of cytoplasmic contents, and noticeable thickening of hyphal cell walls (**Figure 2b2**). Additionally, cell membranes became less distinct, cytoplasmic organelles disintegrated, and massive vacuoles and empty cavities appeared.

### Effect of SCLEO Treatment on Plasma Membrane Integrity

**FIGURE 3:**
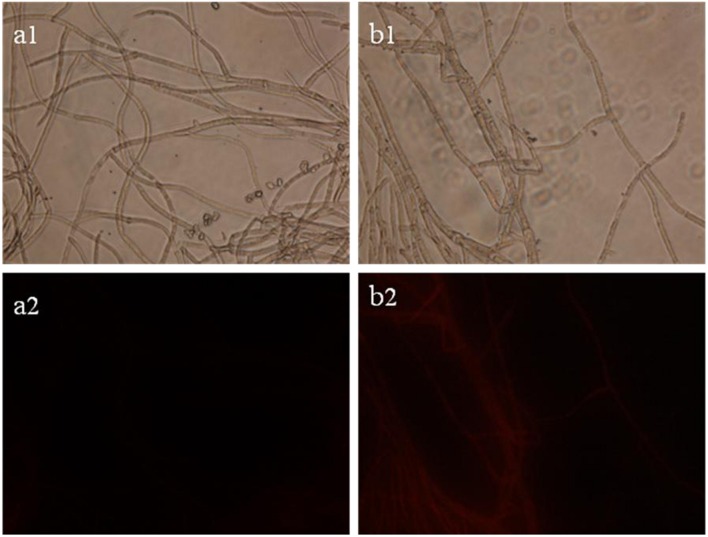
**Images obtained by fluorescence microscopy (×400) for *B. cinerea* treated with SCLEO**. First row: bright field. Second row: propidium iodide (PI). **(a)** Control, **(b)** hyphae treated with SCLEO.

The results of staining *B. cinerea* hyphae with PI are presented in **Figure 3**. Intact hyphae have a slight red background fluorescence (**Figure 3a2**). In contrast, hyphae treated with SCLEO showed markedly higher staining intensity than that of control (**Figure 3b2**), indicating that the integrity of the cell membrane has been compromised and PI readily penetrates the treated hyphae.

### Effects of SCLEO Vapor Treatment on the Quality of Fresh Strawberry Fruit

**FIGURE 4:**
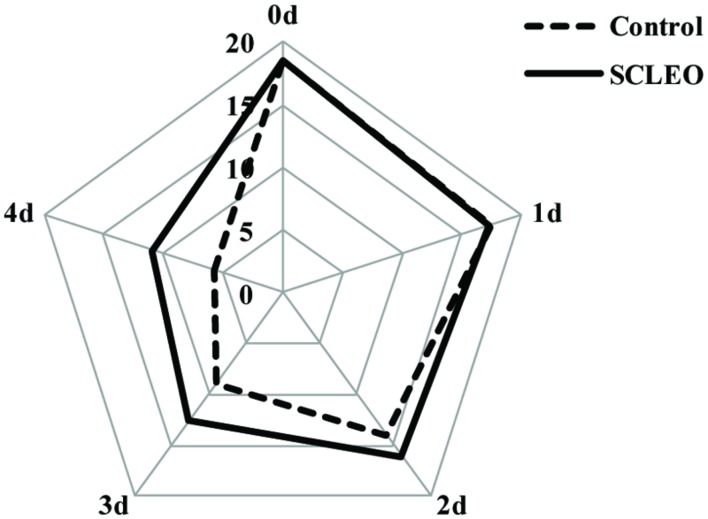
**Effects of SCLEO vapor treatment on the sensory acceptance of fresh strawberries**.

**Table 1** shows changes in quality of fresh strawberry fruits treated with SCLEO. SCLEO treatment did not significantly (*P* > 0.05) affect weight, firmness, TSS, or TA of strawberries during the storage period. However, treatment had significant (*P* < 0.05) effects on the decay index, and decay development was reduced 35 and 25% on the last two days of storage (days 3 and 4). Sensory acceptance scores are plotted on the radar chart shown in **Figure 4**. Scores generally declined during 4 days of storage at 20°C, but treated fruits showed significantly (*P* < 0.05) higher scores from the 2nd day of storage, indicating that SCLEO treatment helps maintain overall quality. Scores for treated fruit were considered good (>10 in a scale of 20) even at the end of the experiment. In this study, a heater was used to promote the volatilization of SCLEO, however, fresh strawberry fruit exposure to more than 0.1 mL/L of SCLEO can cause damage, which appears as fruit softening, pale color and juice leakage.

**Table 1:** Effects of SCLEO vapor treatment on the quality of fresh strawberries during storage.

Treatments	Days of storage				
	0	1	2	3	4
	**Weight loss (%)**				
Control	0.00 ± 0.00^a^	3.11 ± 0.37^a^	6.34 ± 0.24^a^	9.97 ± 0.24^a^	13.69 ± 1.18^a^
SCLEO	0.00 ± 0.00^a^	2.40 ± 0.54^a^	6.58 ± 0.62^a^	9.14 ± 071^a^	15.00 ± 2.53^a^
	**Firmness (N)**				
Control	3.66 ± 0.07^a^	3.77 ± 0.03^b^	3.96 ± 0.26^b^	3.83 ± 0.15^a^	2.96 ± 0.23^a^
SCLEO	3.66 ± 0.07^a^	4.28 ± 0.18^a^	4.57 ± 0.12^a^	3.78 ± 0.19^a^	3.51 ± 0.31^a^
	** TSS (%)**				
Control	14.61 ± 0.70^a^	12.97 ± 0.77^a^	12.57 ± 0.77^a^	12.43 ± 0.60^a^	11.01 ± 0.75^a^
SCLEO	14.61 ± 0.70^a^	12.77 ± 0.80^a^	12.12 ± 0.31^a^	11.23 ± 0.82^a^	11.19 ± 0.85^a^
	** TA(%)**				
Control	0.73 ± 0.03^a^	0.66 ± 0.01^a^	0.63 ± 0.06^a^	0.67 ± 0.02^a^	0.74 ± 0.05^a^
SCLEO	0.73 ± 0.03^a^	0.69 ± 0.03^a^	0.65 ± 0.06^a^	0.64 ± 0.02^a^	0.72 ± 0.08^a^
	**Decay index**				
Control	-	-	-	0.40 ± 0.07^a^	0.66 ± 0.02^a^
SCLEO	-	-	-	0.26 ± 0.04^b^	0.49 ± 0.05^b^

*Values represent means of three independent replicates ± standard error (SD). Values with different superscripts (a vs. b) are significantly different (*P* < 0.05). – indicates no obvious decay development.*

### Effects of SCLEO Treatment on Gray Mold in Artificially Inoculated Strawberries

**FIGURE 5:**
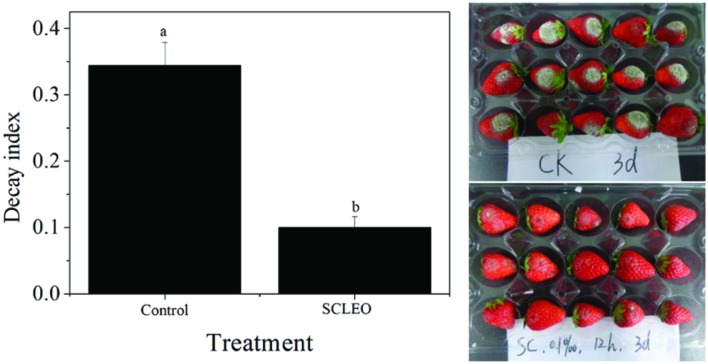
**Effects of SCLEO treatment versus the gray mold decay index in artificially inoculated strawberries**. Vertical bars represent the standard error of the means. Bars labeled with the same letter are not significantly different.

**Figure 5** shows the decay indexes for artificially inoculated strawberries after 3 days of storage at 20°C. The decay index of the control groups was 0.34 that of the treated groups was 0.09. Besides inhibiting the development of decay on the fresh fruit (**Table 1**), SCLEO treatment also reduced 74% of gray mold on the inoculated strawberries.

### Effects of SCLEO Treatment on the Activities of Enzymes Related to Disease Resistance in Artificially Inoculated Strawberries

**FIGURE 6:**
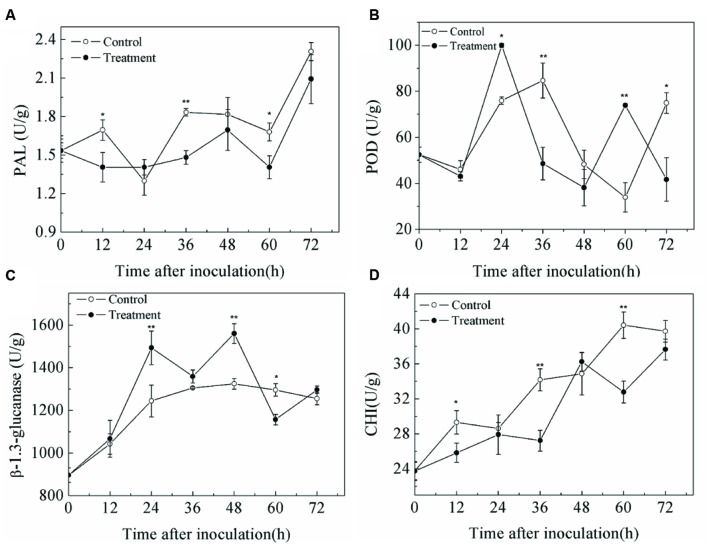
**Effect of SCLEO treatment on phenylalanin ammonia-lyase (PAL) **(A)**, polyphenol oxidase (POD) **(B)**, β-1,3-glutamate **(C)**, and chitinase (CHI) **(D)** activities of strawberries inoculated with *B. cinerea***. Vertical bars represent the SD of the means. ^∗^*P* < 0.05, ^∗∗^*P* < 0.01 based on Tukey’s HSD between control and treated samples at each time point.

Changes of PAL, POD, β-1,3 glutamate, and CHI activities were shown in **Figure 6**. PAL activity of both treated and untreated fruit generally increased after inoculation (**Figure 6A**), but SCLEO-treated fruit had lower PAL activity. POD activity in control and treatment groups showed no obvious pattern, although SCLEO-treated fruit exhibited lower values than those in control samples except at 12 and 60 h (**Figure 6B**). Meanwhile, β-1,3 glutamate activity increased in control fruit in the first 36 h, and then stabilized (**Figure 6C**). SCLEO treatment significantly increased the level of β-1,3 glutamate at 24 and 48 h. CHI activities in treated and untreated fruit increased after inoculation, but SCLEO-treated fruit exhibited lower values except at 48 h (**Figure 6D**).

## Discussion

Several EOs are known to exhibit inhibitory effects on *B. cinerea*, including tea tree oil, oregano oil, thyme oil, and *Tagetes patula* L. oil (Daferera et al., 2003; Romagnoli et al., 2005; Tzortzakis, 2009; Shao et al., 2013a). The reported activities depend on EO composition and especially on the main antimicrobial component. For example, terpinen-4-ol is the principal active component responsible for tea tree oil’s antifungal efficacy against *B. cinerea* (Yu et al., 2015). Daferera et al. (2003) demonstrated that thymol and carvacrol, the main constituents of oregano oil and thyme oil, respectively, have inhibitory effects on gray mold. Piperitonen and piperitenone are the principal constituents responsible for *Tagetes patula* L. oil’s antifungal efficacy against *B. cinerea* (Romagnoli et al., 2005). The main components of SCLEO are α–pinene (59.5%), germacrene (15.2%), limonene (9.7%), β-Pinene (2.8%), and Bornyl acetate (2.0%) (Kalemba and Thiem, 2004). α-pinene is the highest constituent (53.6%) of SCLEO applied in this study, followed by germacrene, limonene and β-Pinene (data not shown), which is almost similar to Kalemba and Thiem (2004). Among of them, α-pinene, limonene and β–Pinene have high activity against *B. cinerea* (Wilson et al., 1997). In this study, SCLEO play effectively antifungal ability to *B. cinerea in vitro* (**Figure 1**) as well as *in vivo* test (**Figure 5**), and α-pinene maybe the principal active component responsible for SClEO’s antifungal ability to *B. cinerea*. Besides, SCLEO also benefit for the quality of strawberry fruits during the shelf life at 20°C (**Table 1**; **Figure 4**). It was suggested that SCLEO vapor is suitable for the postharvest treatment of fresh strawberries stored at room temperature and are able to maintain fruit quality. Shao et al. (2013a) demonstrated that tea tree oil treatment can reduce the decay development on strawberry fruits stored at 20°C. Lemon EO enhanced the chitosan antifungal activity to *B. cinerea* and keep the quality in strawberries at 5°C (Reddy et al., 1998; Perdones et al., 2012), eugenol and citral oil improve shelf-life of strawberries at 0.5°C (Guerreiro et al., 2015). That meanings EOs can play antifungal activities at the different temperature. It was predicted that SCLEO also benefit to the storage-keeping quality of strawberries at lower temperature.

Scanning electron microscopy and transmission electron microscopy are highly effective methods for investigating the changes in microstructural morphology and ultrastructure that occur in microbes exposed to EOs (Soylu et al., 2010; Tao et al., 2014b,c). Soylu et al. (2010) demonstrated that oregano, lavender and rosemary oils can cause considerable morphological degeneration in *B. cinerea* hyphae including cytoplasmic coagulation, vacuolation, hyphal shriveling, and protoplast leakage, and loss of conidiation. Shao et al. (2013b) reported that tea tree oil causes marked myceliar alterations, ruptured plasmalemma and the loss of cytoplasm in *B. cinerea*. Romagnoli et al. (2005) suggested that EO from *Tagetes patula* L. induces alterations in the whole endomembrane system of *B. cinerea*, including degeneration of mitochondrial cristae, release and disintegration of the plasmalemma from the cell wall, and partial dissolution of the nuclear envelope and the rough endoplasmic reticulum. The results reported here show that SCLEO, like other EOs, also significantly alters the hyphal morphology and ultrastructure of *B. cinerea* (**Figure 2**).

Propidium iodide is fluorescent DNA intercalating agent often used to study membrane integrity. It is excluded from intact cells but can enter through membrane lesions and bind to nucleic acid, resulting in red fluorescence (Pinto et al., 2013). The result of SCLEO treated group (**Figure 3**) similar to those obtained in *B. cinerea* treated with tea tree oil (Yu et al., 2015), which generated extensive lesions in the plasma membrane. That are also consistent with the changes in cell membrane and the leakage of internal material observed by TEM (**Figure 2**). Some researchers report that the suppression of fungal growth after treatment with many EOs is associated with alterations in the membrane system and increasing the cell membrane permeability (Romagnoli et al., 2005; Soylu et al., 2006). Dill EOs causes a dose-dependent reduction in ergosterol quantity and the membrane damage of *Aspergillus flavus*, which suggest that plasma membrane is the main target of dill EOs (Tian et al., 2012a,b). Citral treatment also decreased the ergosterol content of *P. italicum* cells and disrupted cell membrane permeability (Tao et al., 2014c). Our previous study showed that tea tree oil destroyed the membrane integrity of *B. cinerea*, consistent with a reduction in total ergosterol content and alteration of the fatty acid composition of the cell membrane (Shao et al., 2013b; Yu et al., 2015). The present study suggests that SCLEO may also act on the composition of the *B. cinerea* cell membrane, resulting in increased membrane permeability and the release of cellular material, which observed by PI staining and SEM, respectively.

*Solidago canadensis* L. essential oil vapor can effectively control the decay development in fresh (**Table 1**) and artificial inoculated (**Figure 5**) strawberry fruits. Control of postharvest diseases by some treatments seems to occur through two different mechanisms: a direct germicidal effect on the pathogen, and an indirect effect resulting from the induction of defense mechanisms in the fruit tissue (Zhao et al., 2008; Liu et al., 2010). Induced resistance mentioned general reference phenylpropanoid metabolic pathway and pathogenesis-related (PR) proteins. PAL is a key enzyme in the first step of the phenylpropanoid pathway, which is involved in the synthesis of phenolic acid and lignin and is important in secondary plant metabolism (Dixon et al., 2002). POD is involved in the last step of monolignol polymerization to form lignin and is directly involved in the induction of defense mechanisms (Passardi et al., 2004). CHI, a member of the PR-2 family, and β-1,3-glucanase, a member of the PR-8 family, are the best characterized PR proteins associated with defense responses (Van Loon et al., 2006). There is compelling evidence that β-1,3-glutamate can act directly by degrading the pathogen cell wall, or indirectly by releasing oligosaccharide elicitors of defense reactions, both of which are potential defense mechanisms against fungal infection (Zhao et al., 2008; Liu et al., 2010). CHI can also degrade chitin, one component of the pathogen cell wall. Although EOs play directly antifungal ability, there are some contrary results about the effects of EOs on the disease resistance of fresh fruits. Thyme oil and tea tree oil vapor treatment can induce the activities of PAL, POD and β-1,3-glucanase in strawberry fruits and avocado fruit during the first period of incubation (Sellamuthu et al., 2013; Shao et al., 2013a). However, octanal treatment does not increase PAL activity and inhibits POD activity (Tao et al., 2014a). Arrebola et al. (2010) also suggested that lemongrass oil treatment does not increase total phenol content or β-1,3-glucanase, CHI, and PAL activities in naturally infected peaches. In this study, although SCLEO treatment may have increased β-1,3-glucanase activity at a few time points, in general this treatment did not significantly increase the activity of disease resistance-related enzymes (**Figure 6**), which suggest that SCLEO treatment does not improve disease resistance in strawberries.

## Conclusion

*Solidago canadensis* L. essential oil exhibited high antifungal activity against *B. cinerea in vitro*, reduced decay development in fresh strawberry fruits, and successfully controlled gray mold in artificially inoculated strawberries. SCLEO vapor treatment also resulted in higher sensory acceptance of strawberries during storage, but did not induce enzymes related to disease resistance in fruit. It was propose that SCLEO-mediated disease inhibition occurs as a result of direct interactions with the fungus itself, and that SCLEO vapor treatment is a potential alternative to synthetic fungicides for the control of phytopathogenic fungi in strawberry fruits. It is also important to reveal the effects of SCLEO on the strawberry fruits stored at different temperature and evaluate the cost of SCLEO application in future.

## Author Contributions

SL and XS designed the experiments. SL, YW, and YL performed the experiments. FX and HW analyzed the data. SL, XS, and HW drafted the manuscript. All authors read and approved the final manuscript.

## Conflict of Interest Statement

The authors declare that the research was conducted in the absence of any commercial or financial relationships that could be construed as a potential conflict of interest.
